# Risk scores to guide the use of anticoagulant in atrial fibrillation

**DOI:** 10.2478/abm-2025-0008

**Published:** 2025-04-30

Atrial fibrillation (AF) is a common arrhythmia worldwide. It is associated with increased morbidity and mortality [1], largely due to an increased risk of ischemic stroke [2]. Anticoagulation is the main strategy to prevent ischemic stroke [3].

AF can lead to blood stasis within the left atrial appendage, resulting in a hemodynamic instability, fibrin formation, and platelet activation. Thus, patients with untreated AF have an annual stroke risk of 5% [4]. Warfarin can inhibit vitamin K epoxide reductase, reducing the synthesis of clotting factors II, VII, IX, and X. Warfarin has been the mainstay of anticoagulant for almost 50 years before newer agents have been developed and used [5]. Warfarin is still widely utilized in many settings [6]. Due to its narrow therapeutic index, the use of warfarin requires a careful balance between thromboembolic protection and bleeding risks from overdose. Scoring systems such as the CHA_2_DS_2_-VASc for stroke prediction and HAS-BLED/HEMORR^2^HAGES for bleeding risk assessment have been evaluated and widely used to manage risk of stroke and prevent bleeding in AF [7]. The SAMe-TT2R2 score has been evaluated to assess the time within the therapeutic range (TTR) and the achievement of optimal international normalized ratio (INR) [8]. The percentage of time in achieving an INR between 2.0 and 3.0 when an anticoagulation is prescribed is desirable [9]. The SAMe-TT_2_R_2_ score plays complementary roles in guiding anticoagulation therapy. Certain guidelines recommend the use of warfarin based on the SAMe-TT_2_R_2_ score. Therefore, the SAMe-TT_2_R_2_ score plays a crucial role in identifying patients who would derive significant benefits from warfarin compared with non-vitamin K antagonist oral anticoagulants (NOAC), lately called direct oral anticoagulants (DOACs).

In this issue, Methavigul K and Krittayaphong R reported another novel risk score model for predicting the poor anticoagulation control in patients with AF taking warfarin. Based on an extensive analysis of SHOB-D_2_AF score in 2,233 patients taking warfarin, they found 1,432 patients having poor anticoagulation control (TTR < 65%), while 801 patients exhibiting good anticoagulation control (TTR ≥ 65%). Symptomatic AF, diabetes, heart failure, and a history of bleeding were associated with an increased risk; whereas, obesity, AF duration, and paroxysmal AF were correlated with a decreased risk of poor anticoagulation control. They use C-statistics to develop a new SHOB-D_2_AF risk score model, which was proposed to be better than the SAMe-TT_2_R_2_ score in predicting poor anticoagulation control [10].

Although the SHOB-D_2_AF risk score is more effective than previous scores in predicting anticoagulant control, its validity must be confirmed through external validation in populations beyond the cohort used for its development. This step is necessary before determining its distinct role in preventing ischemic stroke in patients with AF.
